# Health Mindset and One Year Outcomes in Adult Peritoneal Dialysis (PD) Patients

**Published:** 2024-08-21

**Authors:** Rachel B. Fissell, Marcus G. Wild, David Schlundt, Devika Nair, Ebele M. Umeukeje, Claudia Mueller, Andrew Guide, Robert Greevy, Kerri L. Cavanaugh

**Affiliations:** 1 Division of Nephrology, Vanderbilt University Medical Center, USA; 2 Department of Psychology, Vanderbilt University, USA; 3 Stanford University School of Medicine, USA; 4 Department of Biostatistics, Vanderbilt University Medical Center, USA

**Keywords:** Peritoneal dialysis, Hemodialysis, Health mindset scale, One-way ANOVA, Coronary artery disease

## Abstract

**Background:**

Many patients who start peritoneal dialysis (PD) transition to hemodialysis (HD) after a PD-related complication. Patient psychological factors may influence clinical outcomes. One possible factor is health mindset, or patient belief that their health knowledge and ability can change. The goal of this study is to evaluate the longitudinal associations of baseline health mindset with patient outcomes after one year.

**Methods:**

The Health Mindset Scale (HMS, score 3-18) was administered on paper during clinic to a convenience sample of 100 adult PD patients, to quantify patient mindset along a continuum from fixed mindset (lower scores) to growth mindset (higher scores). Participants were 31% African American, 4% Hispanic, and 64% White American. Demographic and comorbid information were abstracted from medical records. Outcomes assessed at 1 year were death, transition to HD, renal transplant, and maintaining PD.

**Results:**

HMS scores were highest in patients who subsequently received a renal transplant (mean 15, SD 2.1), indicating a growth mindset. HMS scores in patients who died were lower (mean 10, SD 5.2) suggesting a more fixed mindset. Among those who maintained PD, HMS scores were between fixed and growth mindset (mean 12.8, SD 4.2) and similar to those who transitioned to HD (mean 13, SD 4.2). One-way ANOVA for difference in HMS scores by clinical outcome was p = 0.042.

**Conclusions:**

This initial longitudinal study suggests associations between mindset and clinical outcomes. The HMS is a novel and easily administered instrument that quantifies one patient psychological component that could contribute to patient outcomes, and that could also be modified. The HMS may identify individuals who could benefit from specific interventions to favor a growth mindset, with the goal of supporting optimal clinical outcomes.

## INTRODUCTION

PD modality success depends on consistent daily task execution by patients, in their homes, without medical personnel present. Safe, dependable task performance relies on mental skills such as cognitive abilities to perform meticulous sterile technique, trial-and-error problem solving [1], and the resilience to recover from a complication [2,3]. Mindset is a psychological factor that could further influence patient behavior and long-term PD success. Mindsets are beliefs or assumptions that affect individual perceptions and behaviors [4,5]. Mindset theory describes two general categories of mindset: fixed and growth. A fixed mindset is the belief that knowledge and ability cannot develop or improve, because a person’s capacity to learn new skills is related to their inherent personality and innate talent. In contrast, a growth mindset is the belief that knowledge and ability can grow and change with time and effort. Growth mindset theory was initially developed in educational settings, where evidence suggests it supports learning, especially after a student has failed at a difficult task [6]. A growth mindset has been associated with improved health behaviors in Type 1 diabetes [7], pediatric chronic headaches and migraines [8], and physical activity and body mass index in college students at risk for cardiovascular disease [9].

However, the impact of health mindset on clinical outcomes in PD patients is currently unknown. A health mindset that trends toward growth may support initial PD training, recovery from a complication, and achieving a transplant. Our previous work demonstrated variation in health mindset as measured by the Health Mindset Scale (HMS) in a sample of 100 adult PD patients [10]. The variation seen previously in health mindset in PD patients may be attributable to several different factors. Patient upbringing, religious beliefs, underlying disease processes, level of social support, experiences within the health care system, and socioeconomic status could all contribute to baseline mindset. This study reports one year follow-up of the initial sample, to test the hypothesis that greater growth mindset at baseline as quantified by the HMS, is associated with greater frequency of remaining on PD or receiving a renal transplant, and a more fixed mindset at baseline is associated with transition to HD, or with death.

## METHODS

### Study population

We enrolled 101 incident and prevalent PD patients from our institution’s home dialysis unit from April 2019 to June 2020. Despite the COVID-19 pandemic, participants were able to complete study tasks during in-person appointments. Participants were enrolled sequentially from all eligible patients, as scheduling allowed. Participants were adults ages ≥ 18. Participants spoke fluent English with the exception of one patient, who had a caregiver present to translate. Our institution’s Review Board approved all study procedures prior to participant enrollment. Participants provided written informed consent and did not receive monetary compensation.

### Data collection

The principal investigator or a trained research assistant administered survey measures with a paper questionnaire during an in-person encounter. Baseline demographic information, comorbid diseases, and clinical outcomes during the first year after enrollment were abstracted from the electronic medical record. Participant race and ethnicity were included because they associated most often through structural factors to health outcomes. Dialysis vintage was calculated as time since the peritoneal dialysis catheter was placed to date of enrollment. Cardiac disease included coronary artery disease and decreased left ventricular function. Coronary artery disease was coded as present if the patient had a positive cardiac catheterization, or exercise, thallium, or dobutamine stress test, prior to enrollment. Participants who had undergone percutaneous coronary intervention or coronary artery bypass to address coronary artery disease were coded as having coronary artery disease, even if the procedure was successful. Patients with moderate to severe left ventricular dysfunction with an EF < 40% by echocardiogram were coded as having decreased left ventricular function, and classified as having cardiac disease. The comorbid conditions of depression and/or anxiety were assessed by chart review for three variables: past medical history of depression and/or anxiety, clinician note during the study period confirming depression and/or anxiety disorder, and prescription of a medication for depression and/or anxiety during the study period. Patients were classified as having depression and/or anxiety if two out of three variables were present. Patients taking an anti-depressive medication given for a reason other than depression and/or anxiety, such as bupropion for smoking cessation, were not classified as having depression and/or anxiety. Albumin is given as the average of the first two values within 3 months after enrollment.

Transition from PD to HD, commonly called PD drop out, was confirmed if a participant did not return to PD after two months of transition to HD. Based on chart review, the primary reason for PD drop out was classified as either technical and related to PD, or clinical and not related to PD [Table 2]. Examples of reasons for PD drop out that were classified as technical and related to PD were difficulty with eating and sleeping on PD, persistent abdominal pain associated with the PD catheter for unclear reasons, peritonitis, or difficulty draining. Examples of reasons for PD drop out that were classified as clinical issues not related to the PD catheter were a new stroke, progressive dementia, or post-operative death following a major surgery.

To assess mindset related to health, patients were surveyed using a previously employed Health Mindset Scale (HMS) to determine their location on the spectrum from a fixed to growth mindset of health. The HMS, formerly called the Health Belief Scale, is a 3-item Likert-based scale derived from the more general original mindset assessment instrument developed by Carol Dweck and colleagues in the domains of intelligence, personality, and moral character [4]. Participants were instructed to report their agreement with each of the statements about health on a six-point Likert Scale (ranging from ‘strongly agree’ to ‘strongly disagree’). These statements asked patients to assess whether they believed they could change their basic health. The responses were added and analyzed on a scale ranging from 3 to 18, with higher numbers indicating greater growth mindset [10]. Participants’ health literacy [11,12], or their confidence in understanding written and verbal health information, and health self-efficacy [13], or their belief that they have the capacity to manage their health condition(s), were included as conceptually related psychological constructs and assessed with validated self-report measures.

### Statistical analysis

Patients were followed for one year of peritoneal dialysis, or until one of three clinical outcomes occurred: death, transition from PD to HD, or kidney transplant [Table 1]. Results are presented as mean and standard deviation (SD) for numerical variables, and prevalent percentages for each categorical variable, for the overall sample and within each clinical outcome. To benchmark the health of the sample, comorbid disease was quantified using a scale previously validated in PD patients [14]. Spearman’s correlations were used to assess associations between continuous variables and comorbidities. Chi-squared tests were used to test for associations between categorical variables and clinical outcomes. One-way ANOVA examined associations between continuous variables and clinical outcomes. Pairwise comparisons for the one-way ANOVA were not done due to the small sample size. However, to further explore associations between HMS scores and outcomes, HMS scores were regressed on the clinical outcome groups. Statistical tests were performed using R version 4.0.1.

## RESULTS

From 174 eligible PD patients screened between April 2019 and June 2020, 35 were not eligible because they were less than 18 years old, 2 patients refused to participate because of concerns about participating in any clinical trial, one patient was screened and not included because of ongoing severe psychiatric disease, and thirty-five were not approached because of scheduling constraints. The remaining 101 patients were enrolled. One patient subsequently left the study for unclear reasons after enrollment and was removed from the sample. The average age for the sample was 51.8 years (SD 17.0, range 19-86). Diabetes (DM) was present at baseline in 46.0% of the sample. Hypertension (85.0%) and cardiac disease (32.0%) were also prevalent. Almost one quarter of the sample were 90-day incident patients (23%). Dialysis vintage at enrollment ranged from 2 days to 96 months, with an average of 571 days (SD 584) [Table 1].

Average follow-up time for the entire sample was 300 days (SD 112, range 3-366). Twelve patients died, 12 patients transitioned from PD to HD, 10 patients received a renal transplant, and 66 patients remained on PD. There were no statistically significant differences in age, gender, race, or vintage, between the four outcome groups. Spearman’s correlations showed no significant associations between HMS scores and diabetes, hypertension, cardiac disease, history of malignancy, or depression and/or anxiety. There were significant differences in the distribution of comorbid conditions and average serum albumin between the four outcome groups [Table 1]. The patients who received a renal transplant had less cardiac disease and a higher average albumin. The average health literacy score was 12.5, SD 2.7, and there was no significant difference in health literacy score by clinical outcome. The average health self-efficacy score was 13.3, SD 3.3, and there was no significant difference in health self-efficacy score by clinical outcome.

### Mindset and Clinical Outcomes

**Figure 1:**
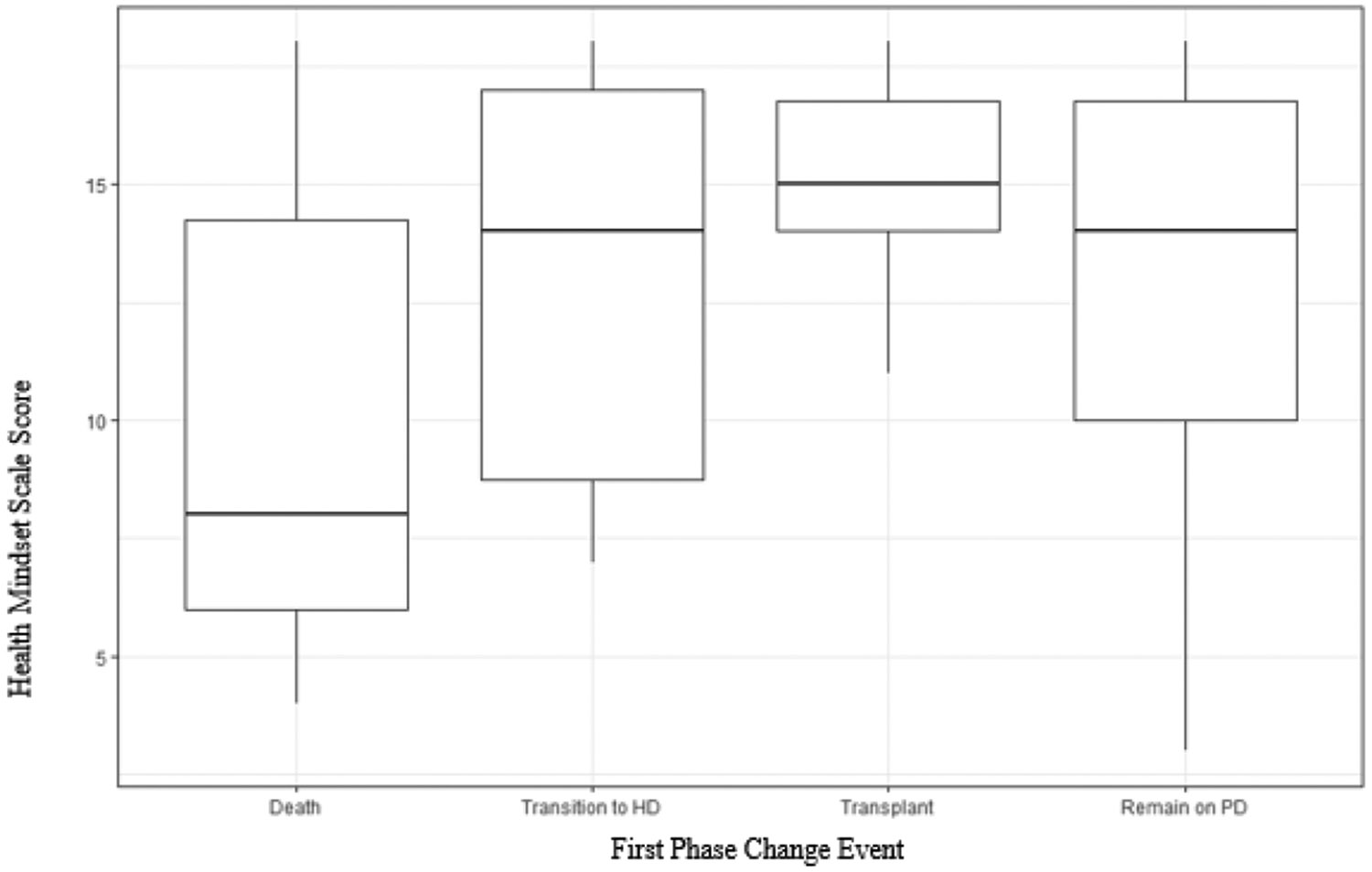
Boxplot of Mean Health Mindset Score by Clinical Outcome, with 25th and 75th Quartiles.

Average baseline HMS scores were different by outcome (one-way ANOVA p=0.042) [Table 1]. Baseline HMS scores were highest in patients who subsequently received a renal transplant (15, SD 2.1), indicating a growth mindset. HMS scores in patients who died were lower (10, SD 5.2) suggesting a more fixed mindset. Among those who maintained PD, HMS scores were between fixed and growth mindset (mean 12.8, SD 4.2) and similar to those who transitioned to HD (mean 13, SD 4.2). To further explore these associations, HMS scores were regressed on the clinical outcome groups [Table 3] and shown as a boxplot in Figure 1. The R^2^ for this regression is 0.08, with lower HMS scores significantly associated with death.

In terms of PD drop out, twelve patients transitioned from PD to HD during one year of follow-up [Table 2]. Seven patients transitioned from PD to HD for a reason related to PD, and only one of those was related to peritonitis. For the five patients that stopped PD for a clinical reason unrelated to PD, the average HMS score was 14.2, SD 3.4. The average HMS in the seven patients who stopped PD for a PD related reason was lower, at 12.1, SD 4.7.

## DISCUSSION

The primary finding of this study is variation in 1-year clinical outcomes by baseline HMS. Consistent with our hypothesis, average baseline HMS scores were highest in patients who received a renal transplant at one year of follow-up, and lowest in patients who died. A lower baseline HMS was also present in patients who were less healthy in general, as indicated by a greater prevalence of cardiac disease and lower average albumin. In addition, a promising exploratory finding was that the average HMS score was lower in the seven patients who transitioned from PD to HD for a PD-related reason, as compared to the five patients who transitioned to HD for a clinical reason unrelated to PD.

Despite a relatively small sample, with only 1-year of follow-up time, baseline growth mindset was notably higher in patients who subsequently achieved a renal transplant. This is consistent with previous literature on the psychosocial factors that can impact dialysis patient outcomes. Renal transplant evaluation and then listing requires multiple appointments and tests. A recent prospective cohort of kidney transplant candidates found a high prevalence of depressive symptoms, and that patients with depressive symptoms were less likely to be listed [15]. Depression may interfere with task completion. In contrast, a growth mindset supports persistence at difficult tasks. A growth mindset could contribute to successful navigation of the transplant process. Patients and their families need to have a fundamental belief that they can successfully complete tasks to change from unlisted to listed, maintain eligibility while on the waitlist, and then receive and manage a kidney transplant. Similarly, sustaining PD requires the ability to consistently execute treatments and handle complications. So, it also makes sense that lower HMS scores suggesting a more fixed mindset, were present in patients who transitioned from PD to HD because of a technical reason related to their home dialysis treatments. Patients with a more fixed mindset may have greater difficulty with the trial-and-error process that is often needed to navigate PD challenges and find solutions [1].

Other important emotional and behavioral factors that affect PD outcomes include patient burnout, or health-behavior-related chronic stress that goes unmanaged, and depression. Patient burnout may precede PD technique failure [3,16]. Depression has also been associated with peritonitis [17]. A recent multicenter observational study specifically shows associations between depression and impaired cognition in patients receiving PD, two factors that can negatively impact performance of sterile technique [18]. Depression and anxiety were prevalent in this sample. The actual prevalence may be higher, since assessment was made by chart review, not by administration of an instrument specific to depression and/or anxiety. This is important because depression and anxiety may influence baseline mindset. The presence of untreated depression and/or anxiety may also impair patient capacity to shift from a more fixed mindset to a more growth oriented mindset, in response to guidance from the care team or other intervention. Patient psychological challenges are different from other patient risk factors for PD drop out and death such as older age and high peritoneal membrane transport status, because they can be modified. Depression in dialysis patients can be treated [19] and patient burnout can be reduced. For example, tailoring the PD prescription to enable days off and give patients and their families respite from the daily, relentless PD regimen supports sustained PD [20]. Each of these strategies requires a willingness on the part of patients, their families, and dialysis providers to try a new approach and then discard that approach if it is unsuccessful, characteristics of a growth mentality. Country, culture, and facility variation in peritonitis outcomes [21] and PD time on therapy [22] could be partially explained by differences in approach and resources allocated to the psychological aspects of sustained PD.

Modifying patient behavior to reduce PD drop out may be about more than identifying and addressing psychological barriers. It may be just as important to augment and amplify positive coping skills such as resilience, motivation, and a growth mindset. Several small but promising randomized controlled trials have successfully increased mindset to improve outcomes in health care settings other than PD, sometimes as an addition that augments the effectiveness of other interventions [6,23,24]. A growth mindset is also aligned with theory supporting patient activation. Patients who believe that they can grow and change, may be more likely to take an active, engaged, and effective role in their own health care [25,26].

Peritonitis and other catheter complications may precede PD drop out [2,27]. Efforts to reduce PD drop out often focus on training and retraining patients in PD catheter management. However, the effects of training regimens on PD patient outcomes are poorly understood, and the best training practices are still being defined. A recent observational study enrolling patients from seven countries found no associations between peritonitis risk and when, where, how or how long PD patients were trained [28]. Two large, well-designed randomized controlled trials testing training strategies to prevent peritonitis did not significantly decrease all-cause peritonitis rate or reduce transfer to HD [29,30]. However, these and other current interventions focus on patient training and education regarding the procedures of PD without addressing key psychological factors that may impact PD success. Protocols designed to reduce peritonitis and PD drop out that are informed by a biopsychosocial conceptualization of PD, with more focused and robust psychological interventions, may more effectively support long term PD maintenance.

The primary limitation of this exploratory study is the small number of patients and affiliated outcomes during the study period. Another major limitation of this study is classification of depression and/or anxiety by chart review, instead of by an instrument administered at the start of the study period. Since lower baseline HMS is associated with both higher illness burden and worse outcomes, a multivariate model with adjustment for comorbid conditions is of interest. A longer duration study may be warranted for evaluation of these important outcomes over time.

Healthcare mindset may be a key predictor of desired clinical outcomes among patients receiving PD for end-stage kidney disease. This is important because unlike clinical indicators such as age or cardiac disease, mindset can potentially be modified. Our study supports previous work showing a need for improved support of PD patients beyond technical management of a clinical issue. Additional studies are needed to learn whether support for beneficial patient psychological elements such as motivation, tenacity and persistence, and perhaps a growth mindset could potentially help patients to relearn sterile technique after a gram-positive peritonitis, weather a visit to the ER or a hospitalization, and then continue on PD without a transition to HD. Larger studies are needed to disentangle the differences found in this study in clinical outcomes, and the association between fixed mindset and greater illness burden. However, the presence of these differences suggests that the HMS may be measuring a psychological factor important for survival, and could be useful to identify patients at risk for a poor outcome, who could then receive a targeted intervention. Addressing mindset through novel coaching and educational interventions may be the key to optimizing psychological factors in peritoneal dialysis patients as they undergo training, transition to PD, and then confront complications. Mindset may emerge as a useful and specific concept for intervention to support peritoneal dialysis patients at risk of poor health outcomes.

## Figures and Tables

**Table 1.** One Year Follow-up on Baseline Sample of Adult PD Patients

Clinical Outcome		Overall	Death	PD --> HD	Transplant	Maintain PD	p-value
		N = 100	N = 12	N = 12	N = 10	N = 66	
	Total follow-up time (days) mean (SD)	300 (112)	172 (100)	192 (139)	161 (112)	363 (16)	
Baseline Demographics							
	Age (yrs): mean (SD)	51.8 (17.0)	56.3 (15.9)	51.3 (14.6)	39.2 (14.2)	53.0 (17.3)	0.080
	Gender (male)	57 (57.0%)	9 (75.0%)	7 (58.3%)	5 (50.0%)	36 (54.5%)	0.594
	Race (%)						0.868
	African American	31 (31.0%)	4 (33.3%)	5 (41.7%)	4 (40.0%)	18 (27.3%)	
	Hispanic	4 (4%)	0 (0.0%)	0 (0.0%)	0 (0.0%)	4 (6.1%)	
	Southeast Asian	1 (1%)	0 (0.0%)	0 (0.0%)	0 (0.0%)	1 (1.5%)	
	White American	64 (64.0%)	8 (66.7%)	7 (58.3%)	6 (60.0%)	43 (65.2%)	
	90-day Incident (%)	23 (23.0%)	1 (8.3%)	4 (33.3%)	1 (10.0%)	17 (25.8%)	0.363
	Prevalent (%)	77 (77.0%)	11 (91.7%)	8 (66.7%)	9 (90.0%)	49 (74.2%)	
	Vintage (days): mean (SD)	571 (584)	900 (799)	474 (692)	537 (371)	553 (537)	0.218
Baseline Comorbid Conditions							
	Diabetes mellitus (%)	46(46.0%)	8 (66.7%)	5 (41.7%)	1 (10.0%)	32(48.5%)	0.062
	Hypertension (%)	85 (85.0%)	9 (75.0%)	9 (75.0%)	9 (90.0%)	58 (87.9%)	0.516
	Cardiac disease (%)	32 (32.0%)	8 (66.7%)	2 (16.7%)	0 (0.0%)	22 (33.3%)	**0.004**
	Malignancy (%)	19 (19.0%)	1 (8.3%)	3 (25.0%)	3 (30.0%)	12 (18.2%))	0.578
	Depression/Anxiety (%)	37 (37%)	5 (41.7%)	3 (25%)	3 (30%)	26 (39.4%)	0.762
	Albumin: mean (SD)	3.5 (0.49)	3.0 (0.48)	3.3 (0.44)	3.6 (0.42)	3.6 (0.45)	**< .001**
	Davies Comorbidity Score	1.19 (1.07)	1.92 (1.24)	1.00 (0.85)	0.40 (0.52)	1.21 (1.06)	**0.008**
Psychosocial Measurements							
	Mindset Scale: mean (SD)	12.77 (4.2)	10 (5.2)	13 (4.2)	15 (2.1)	12.89 (4.1)	**0.042**
	Health Literacy: mean (SD)	12.54 (2.7)	11.08 (3.3)	13.58 (2.9)	12.70 (1.8)	12.59 (2.7)	0.159
	Health Self-Efficacy (SD)	13.3 (3.3)	11.6 (3.1)	13.6 (3.8)	13.9 (2.4)	13.4 (3.3)	0.300

**Table 2:** Transition from PD to HD, or PD Drop-out

Patient #	HMS	Davies Comorbid Score	Factors Contributing to PD Drop Out	PD Drop Out Classification
1	7	1 (Mid)	patient request: difficulty with eating and sleeping on PD (cycler), depression noted in the chart	PD, technical
2	7	1 (Mid)	patient request: rapid transporter, fatigue, shortness of breath, no appetite and not eating	PD, technical
3	8	1 (Mid)	patient request: persistent abdominal pain associated with PD catheter, peritonitis ruled out	PD, technical
4	9	1 (Mid)	transitioned to HD after a stroke	clinical, not PD related
5	13	0 (Low)	transitioned to HD after nephrectomy, then died about 2 months after nephrectomy	clinical, not PD related
6	14	3 (High)	peritonitis	PD, technical
7	14	0 (Low)	incomplete draining, progressive volume overload	PD, technical
8	15	0 (Low)	progressive dementia, making PD not feasible	clinical, not PD related
9	17	2 (Mid)	tunnel infection and abdominal cellulitis	PD, technical
10	17	1 (Mid)	bilateral nephrectomies for cancer, planned transition to HD following surgery	clinical, not PD related
11	17	1 (Mid)	finger ulcerations that interfere with dialysis related tasks at home	clinical, not PD related
12	18	1 (Mid)	patient request: difficulty draining with new PD catheter	PD, technical

**Table 3:** Regression of HMS scores on Outcome Group

Variable	Coefficient	p-value
(Intercept)	12.89	
Transition to HD	0.11	0.934
Transplant	2.11	0.133
Death	−2.89	0.027

R-squared: 0.082
